# Subacute Stent Thrombosis Revealing High On-Treatment Platelet Reactivity to Clopidogrel: A Case Report

**DOI:** 10.7759/cureus.106062

**Published:** 2026-03-29

**Authors:** Elias Nabhan, Oberlin Andriamihary, Edouard Naoum Nehme, Alexandrina-Paula Vana, Nabil Poulos

**Affiliations:** 1 Cardiology, Centre Hospitalier Josephine Baker, Gonesse, FRA

**Keywords:** clopidogrel resistance, high on-treatment platelet reactivity, percutaneous coronary intervention, stent thrombosis, vasodilator-stimulated phosphoprotein assay

## Abstract

Stent thrombosis (ST) is a severe complication of percutaneous coronary intervention (PCI), often presenting as acute myocardial infarction or sudden death. We present a case of a 51-year-old male patient with a history of hypertension and dyslipidemia who underwent elective PCI with stent implantation in the first marginal branch for the management of exertional angina. Five days post-procedure, he presented with an inferior ST-segment elevation myocardial infarction (STEMI). Urgent coronary angiography demonstrated subacute thrombosis of the circumflex artery and the ostium of the first marginal at the stent site. Platelet function testing using the vasodilator-stimulated phosphoprotein (VASP) index demonstrated high on-treatment platelet reactivity (HPR) to clopidogrel, which guided the decision to switch antiplatelet therapy to ticagrelor. The patient was successfully treated with glycoprotein IIb/IIIa inhibitors and with a switch from clopidogrel to ticagrelor as the P2Y12 receptor inhibitor. Follow-up angiography after 48 hours of glycoprotein IIb/IIIa inhibitor infusion showed complete thrombus resolution with restoration of normal blood flow. This case illustrates that a VASP index exceeding the 60% poor-response threshold may contribute to early ST, and that prompt platelet function testing should guide escalation to more potent P2Y12 receptor inhibitors in patients presenting with unexplained subacute ST.

## Introduction

Percutaneous coronary intervention (PCI) with stent implantation is the mainstay treatment for obstructive coronary artery disease. Dual antiplatelet therapy (DAPT) using aspirin and a P2Y12 receptor inhibitor is mandatory to prevent stent thrombosis (ST), especially with drug-eluting stents (DES) [1]. ST is classified by the Academic Research Consortium (ARC) as acute within 24 hours, subacute from 24 hours to 30 days, late from 30 days to one year, and very late beyond one year [2]. While the use of potent P2Y12 receptor inhibitors like ticagrelor or prasugrel has reduced ischemic events, clopidogrel remains commonly used due to cost and bleeding risk profiles in many populations [3]. A major limitation associated with clopidogrel is its interindividual variable metabolism, which results in a large proportion of patients experiencing high on-treatment platelet reactivity (HPR). The vasodilator-stimulated phosphoprotein (VASP) assay specifically evaluates the degree of P2Y12 receptor inhibition and provides a quantitative measure of platelet reactivity [4]. We present a case of subacute ST associated with HPR to clopidogrel, diagnosed via the VASP assay, highlighting the clinical relevance of platelet function testing in this setting.

## Case presentation

**Figure 1:**
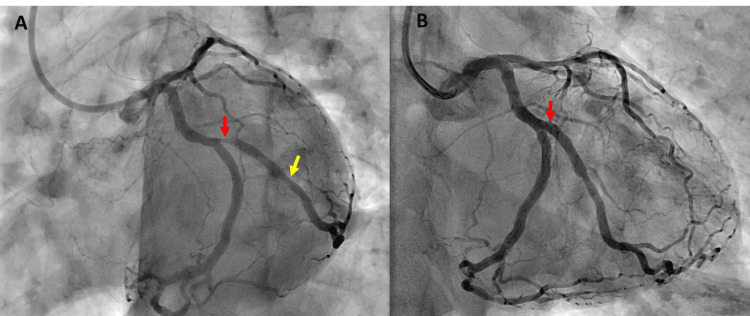
Initial coronary angiography and angioplasty (A) Coronary angiography demonstrates a critical ostial stenosis of the first marginal branch (red arrow) and a less than 50% lesion in the mid-portion of the first marginal artery (yellow arrow). (B) Post-angioplasty result after DES implantation shows successful stent deployment.

A 51-year-old man was referred by his cardiologist for the assessment of exertional angina. His medical history was significant for hypertension and dyslipidemia. He was a non-smoker and denied alcohol consumption. Coronary angiography revealed a significant lesion in the first marginal branch of the circumflex coronary artery. Subsequently, he underwent successful angioplasty with the placement of a 3.0 × 16 mm Firehawk Liberty sirolimus-eluting stent at the ostium of the first marginal artery. The final angiographic result showed good stent apposition with no residual stenosis. Figure 1 shows the initial coronary angiography and angioplasty result. The procedure was uncomplicated, and the patient was discharged on standard DAPT with aspirin 75 mg daily and clopidogrel 75 mg daily.

**Figure 2:**
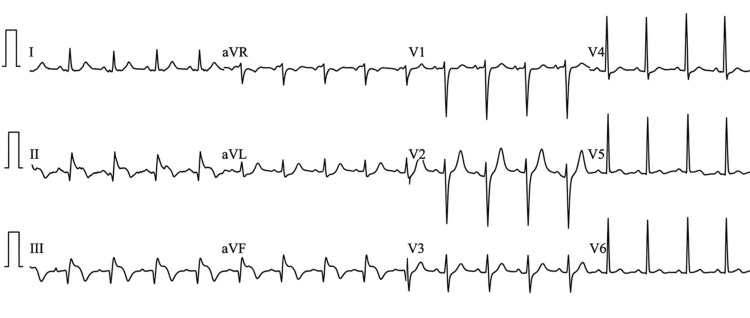
Electrocardiogram at readmission The 12-lead electrocardiogram reveals significant ST-segment elevation in the inferior leads II, III, and aVF, with reciprocal changes in the lateral leads, consistent with an acute inferior STEMI.

Five days post-discharge, the patient presented to the emergency department with severe crushing chest pain. An electrocardiogram (ECG) was immediately performed and revealed significant ST-segment elevation in the inferior leads II, III, and aVF, consistent with an acute inferior ST-segment elevation myocardial infarction (STEMI), as shown in Figure 2.

**Figure 3:**
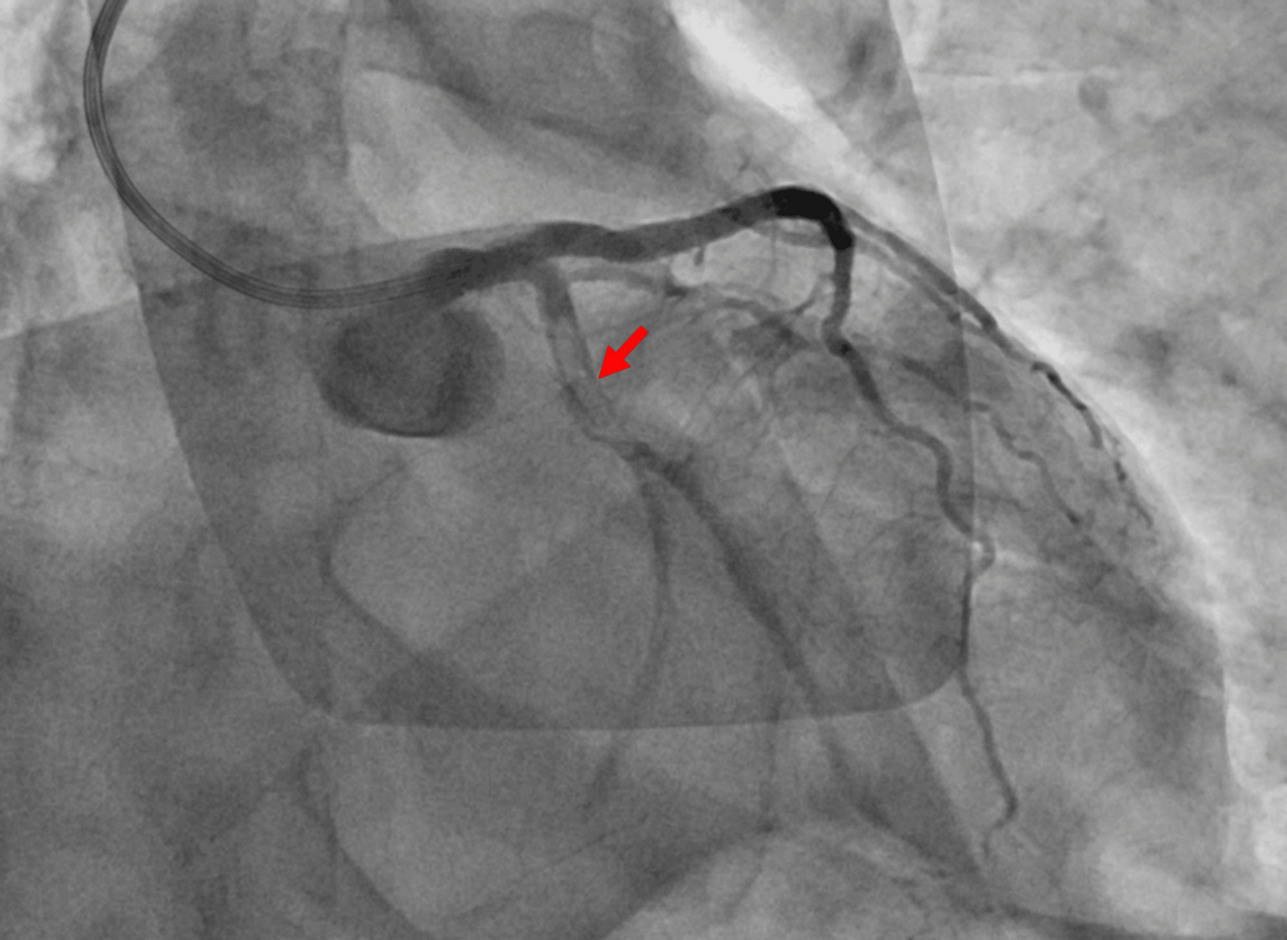
Urgent coronary angiography showing an acute thrombotic occlusion of the proximal circumflex artery (red arrow) and the ostium of the first marginal branch.

An urgent coronary angiography demonstrated subacute thrombotic occlusion of the proximal circumflex artery and the ostium of the first marginal branch at the site of the recently placed DES consistent with subacute ST, as shown in Figure 3.

Adherence to the prescribed antiplatelet therapy was verified based on confirmation from the patient and his wife.

The patient received loading doses of ticagrelor, aspirin, and heparin. Due to the large thrombus burden, the mechanical manual thrombus aspiration resulted in only partial restoration of flow; a glycoprotein IIb/IIIa inhibitor infusion with tirofiban was subsequently started for 48 hours. In parallel, platelet function testing for clopidogrel resistance was performed using the VASP assay, a flow cytometry-based method.

The results of platelet function testing are presented in Table 1, which shows a VASP index of 83%, interpreted as a poor response and indicative of HPR. A VASP platelet reactivity index of 50% or greater is the consensus-defined threshold associated with an increased risk of ischemic events including ST in patients on clopidogrel [5], while an index exceeding 60% is specifically classified as a poor response according to the assay interpretation criteria used in this case (BioCytax-MAVIOS). This patient's result of 83% exceeded both thresholds, showing a significant HPR to clopidogrel.

**Table 1:** Platelet function testing results. The vasodilator-stimulated phosphoprotein (VASP) assay was performed using BioCytax-MAVIOS on Beckman Coulter equipment. An index greater than 60% indicates poor response to clopidogrel, confirming high on-treatment platelet reactivity (HPR).

Parameter	Result	Reference range
Vasodilator-Stimulated Phosphoprotein (VASP) Index	83%	<50% good response, 50-60% intermediate response, >60% poor response

**Figure 4:**
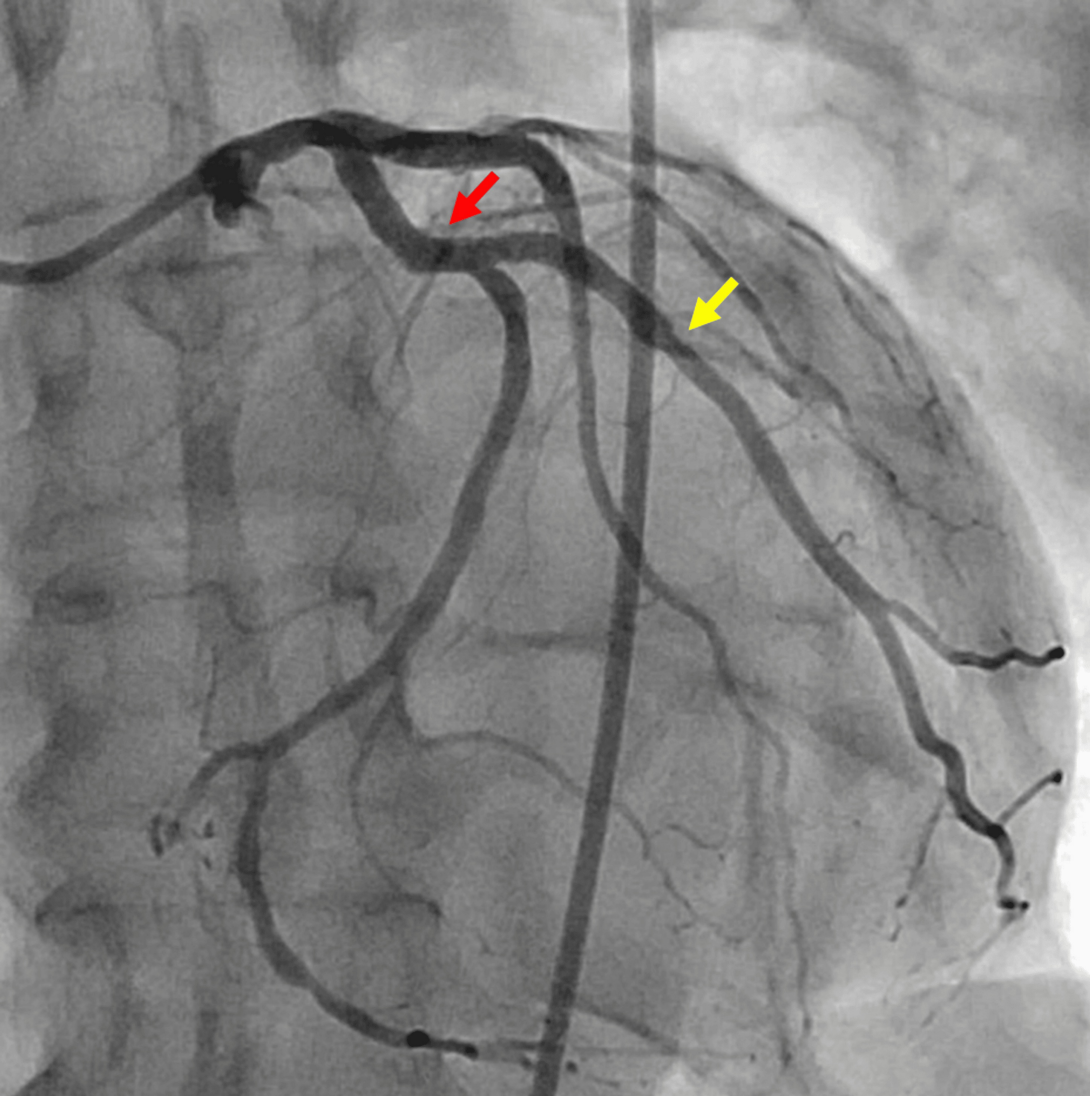
Control coronary angiography post-treatment. Coronary angiography showing complete disappearance of thrombus (red arrow) from the proximal circumflex artery and the ostium of the first marginal stent site. There is no evidence of restenosis, and the previously noted non-significant lesion in the mid-first marginal artery remains unchanged (yellow arrow).

After 48 hours of glycoprotein IIb/IIIa inhibitor infusion and after switching antiplatelet therapy, a control coronary angiography along with intracoronary optical coherence tomography showed complete disappearance of the previously described thrombus and excluded a mechanical etiology such as stent underexpansion or malapposition (Figure 4).

Based on the strong suspicion and subsequent confirmation of HPR with VASP 83%, along with the absence of a mechanical cause of ST, clopidogrel was discontinued and the patient was switched to ticagrelor 90 mg twice daily. The patient's subsequent clinical course was uneventful, and the follow-up angiogram confirmed the complete resolution of the thrombus with the patient remaining asymptomatic at subsequent clinic visits.

## Discussion

This case demonstrates the significant consequences of HPR associated with clopidogrel therapy. The patient was otherwise compliant with his medications but had a subacute ST five days post uncomplicated PCI.

The VASP assay result of 83% confirmed HPR in this patient, exceeding both the 50% consensus threshold associated with increased ischemic risk [5] and the 60% cutoff defined as poor response by the assay criteria.

Clopidogrel is a prodrug that requires conversion to its active metabolite by hepatic cytochrome P450 (CYP450) enzymes. Genetic polymorphisms most notably loss-of-function alleles CYP2C19*2 and CYP2C19*3, can lead to reduced enzyme function, resulting in insufficient production of the active metabolite and subsequently causing HPR [4]. Studies report a wide range of clopidogrel resistance in the literature (5-40%) based on patient populations and methodologies used to test for resistance [4]. HPR has been linked to an increased risk of adverse events, especially ST [6].

The VASP assay was selected in this case due to its high specificity for the P2Y12 receptor pathway, distinguishing it from whole-blood aggregometry-based methods such as VerifyNow or Multiplate, which may be influenced by other platelet activation pathways [5]. Although there are no strong recommendations for routine platelet function testing per current guidelines, testing may be useful in selected high-risk populations such as unexplained ST [7]. In this patient, the VASP result provided an objective finding supporting the clinical decision to change therapy. The discovery of HPR necessitated a change in therapy, and the patient was switched to ticagrelor. Several large randomized trials comparing potent P2Y12 receptor inhibitors with clopidogrel have demonstrated higher reductions in ischemic events, supporting the decision to use ticagrelor for our patient who had already demonstrated a thrombotic event on clopidogrel [3,8].

This case highlights several key points. First, when a patient presents with early ST, a complete work-up should be undertaken to look for mechanical causes such as stent malapposition or underexpansion via intravascular imaging and biological etiologies through platelet function testing [9]. Second, although platelet function testing is not recommended by current guidelines as a routine test, it may be useful in selected high-risk scenarios such as unexplained ST [7]. Third, the presence of documented HPR to clopidogrel should be recorded in the patient's medical file as a lifelong important finding to guide future therapy, ensuring they receive appropriate alternative P2Y12 receptor inhibitors.

This case report has several limitations that should be considered. As a single case, the findings may not be generalizable to all patients undergoing PCI. Genetic testing for CYP2C19 polymorphisms was not performed, which could have provided additional mechanistic insight. The decision to perform platelet function testing was made after the thrombotic event. Despite these limitations, the case provides important clinical lessons regarding the management of HPR patients who develop ST.

## Conclusions

This case describes the clinical course of a patient with subacute DES thrombosis associated with clopidogrel-related HPR. It highlights the role that platelet function testing, specifically the VASP assay, can play in evaluating ST. The patient was successfully treated through aggressive acute-phase antithrombotic therapy with a glycoprotein IIb/IIIa inhibitor and a prompt switch from clopidogrel to the more potent P2Y12 receptor inhibitor ticagrelor. This case emphasizes the role of individualized medicine and the need for a high index of suspicion for antiplatelet drug resistance in patients who present with early coronary ST.
